# Driving factors and temporal fluctuation of Collembola communities and reproductive mode across forest types and regions

**DOI:** 10.1002/ece3.3035

**Published:** 2017-05-10

**Authors:** Melanie M. Pollierer, Stefan Scheu

**Affiliations:** ^1^ J.F. Blumenbach Institute of Zoology and AnthropologyGeorg August University GöttingenGöttingenGermany

**Keywords:** forest management intensity, forest soil food web, mode of reproduction, parthenogenesis, soil animals

## Abstract

Despite the major role of Collembola in forest soil animal food webs, ecological and evolutionary determinants of their community composition are not well understood. We investigated abundance, community structure, life forms, and reproductive mode of Collembola in four different forest types (coniferous, young managed beech, old managed beech, and unmanaged beech forests) representing different management intensities. Forest types were replicated within three regions across Germany: the Schorfheide‐Chorin, the Hainich, and the Swabian Alb, differing in geology, altitude, and climate. To account for temporal variation, samples were taken twice with an interval of 3 years. To identify driving factors of Collembola community structure, we applied structural equation modeling, including an index of forest management intensity, abiotic and biotic factors such as pH, C‐to‐N ratio of leaf litter, microbial biomass, and fungal‐to‐bacterial ratio. Collembola abundance, biomass, and community composition differed markedly between years, with most pronounced differences in the Schorfheide, the region with the harshest climatic conditions. There, temporal fluctuations of parthenogenetic Collembola were significantly higher than in the other regions. In the year with the more favorable conditions, parthenogenetic species flourished, with their abundance depending mainly on abiotic, density‐independent factors. This is in line with the “Structured Resource Theory of Sexual Reproduction,” stating that parthenogenetic species are favored if density‐independent factors, such as desiccation, frost or flooding, prevail. In contrast, sexual species in the same year were mainly influenced by resource quality‐related factors such as the fungal‐to‐bacterial ratio and the C‐to‐N ratio of leaf litter. The influence of forest management intensity on abundances was low, indicating that disturbance through forest management plays a minor role. Accordingly, differences in community composition were more pronounced between regions than between different forest types, pointing to the importance of regional factors.

## 1 Introduction

Understanding the structure and functioning of soil animal communities has been one of the long‐standing challenges for soil ecologists. Belowground communities are remarkably diverse and play a major role for ecosystem functioning (Bardgett & van der Putten, 2014). However, it is difficult to disentangle ecological determinants of community composition, such as resource availability and quality, competition, regional and climatic differences, and differences in reproductive strategies. Additionally, the relative importance of these structuring mechanisms may underlie temporal fluctuations.

Collembola are among the most abundant and species‐rich taxa in forest soil animal food webs, reaching similar high biomasses as Oribatida and Enchytraeidae in beech and spruce forests (Schaefer, 1990; Scheu et al., 2003). They occupy a wide spectrum of trophic niches (Chahartaghi et al., 2005); their distribution along a gradient of vertical stratification has been linked to morphological characteristics, such as pigmentation, length of furca, and body size, resulting in so‐called eco‐morphological groups or life forms (Christiansen, 1964; Rusek, 2007). Similar to other abundant soil animal taxa such as Oribatida, Lumbricidae, Enchytraeidae, and Nematoda, Collembola comprise species with parthenogenetic reproduction (Chahartaghi, Scheu, & Ruess, 2006; Chernova et al., 2010). The underlying mechanisms and causes that determine the distribution and patterns of sexual vs. parthenogenetic reproduction are widely debated (Tagg, Innes, & Doncaster, 2005; Meirmans 2009; Neiman, Sharbel, & Schwander, 2014). Presumably, multiple mechanisms are at work that outweigh the twofold cost of sex, among them genetic variation in offspring by the introduction of new gene combinations, the spread of advantageous mutations, and the removal of deleterious mutations (West, Lively, & Read, 1999). Further, the importance of resources (structure and availability) in determining the success of sexual versus parthenogenetic reproduction is becoming increasingly recognized (Scheu & Drossel, 2007; Song, Drossel, & Scheu, 2011). Typically, sexuals are superior to parthenogens as they are better in exploiting new and unused resources. In soil, detritus as the main resource is abundant and this may favor parthenogenetic species as it is never exploited fully (Scheu & Drossel, 2007). Additionally, biotic factors such as resource quality and availability may be superimposed by abiotic factors, for example, when environmental conditions are harsh. Harsh abiotic conditions may lead to an advantage of parthenogenetic reproduction, as in the case of geographic parthenogenesis (Song et al., 2011). However, intense abiotic stress in extreme habitats such as metal‐polluted sites (Gillet & Ponge, 2003) or seashores (Niklasson, Petersen, & Parker, 2000) may lead to the prevalence of sexuals in otherwise parthenogenetic populations, presumably because sexual species are better able to select for favorable genes when faced with stressed environments on intermediate time scales. At small spatial scales, temporal fluctuations of environmental conditions may contribute to sustaining both sexual and asexual populations in forest soils.

Abundance and species composition of Collembola can be linked to both biotic and abiotic factors. From a trophic perspective, Collembola form a continuum from herbivores to primary and secondary decomposers (Chahartaghi et al., 2005), with microorganisms, in particular fungi (Ruess et al., 2007), forming a major food source, to predators consuming, for example, nematodes (Heidemann et al., 2014). Additionally, many Collembola feed on humified organic matter including animal feces enriched in bacteria (Anderson & Bignell, 1980; Ponge, 2000) which also may form part of the food of Collembola (Haubert et al., 2006; Pollierer et al., 2012). In forests, land‐use and management practices have been shown to affect microbial community composition and the relative strengths of the fungal and bacterial energy channel (Pollierer, Ferlian, & Scheu, 2015), presumably impacting Collembola community composition (Filser et al., 2002). Additionally, Collembola community composition is linked to vertical stratification (Berg, Kniese, & Verhoef, 1998; Salmon et al., 2014). Collembola life forms differ in habitat layer and presumably exploit different resources, thereby playing different ecosystem roles (Berg & Bengtsson, 2007; Potapov et al., 2016). Land‐management practices may have differing effects on Collembola life forms. Salamon, Scheu, and Schaefer (2008) found increased Collembola diversity with forest age, especially in epedaphic species which presumably benefitted from the more pronounced herb layer in older forests, providing more diverse food resources. In contrast, hemiedaphic species in the latter study were mainly influenced by soil pH and soil water content. Abiotic factors, such as soil chemistry and humus form (Hågvar & Abrahamsen, 1984; Loranger et al., 2001), as well as temperature and moisture (Petersen, 2011) can exert strong effects on Collembola community composition. In Oribatida, these predominantly regional factors, rather than different forest types, mainly affected community composition (Erdmann, Scheu, & Maraun, 2012). However, thickness of the litter layer that varies with forest type and management intensity can also have major effects on abundance (Badejo, Nathaniel, & Tian, 1998) and community composition (Osler, Korycinska, & Cole, 2006) of soil mesofauna. Additionally, stability of Collembola communities is closely related to environmental conditions (Chernova & Kuznetsova, 2000) and therefore may depend on between‐year variation of climate and associated abiotic factors such as moisture and temperature. Bengtsson (1994) suggested that forest soil fauna communities vary little between years unless they are subject to substantial environmental impact such as forest management. Therefore, interannual fluctuations in Collembola community composition may differ between forest types.

To investigate major determinants of Collembola community composition, we sampled and identified Collembola species from four different forest types (coniferous, young and old managed beech, and unmanaged beech) across three regions in Germany, the Swabian Alb in the southwest, the Hainich in the center, and the Schorfheide in the northeast. The regions were chosen because they span a broad geographic range within Germany and differ in geology, altitude, and climate, with the Schorfheide featuring the harshest conditions in terms of climate (continental) and geology (Fischer et al., 2010). To account for temporal variation, samples were taken twice, in the same season (spring), but with an interval of 3 years. To determine the relative importance of drivers impacting total abundance and reproductive mode, we applied structural equation modeling.

We hypothesized that Collembola are more abundant in coniferous forests due to a more pronounced litter layer providing more habitat structure. We assumed that Collembola rely more on fungal than on bacterial diets and therefore abundances to be higher in unmanaged beech forests as compared to managed beech forests due to higher fungal‐to‐bacterial ratios (Pollierer et al., 2015). We expected epedaphic Collembola to be more strongly affected by management intensity than species inhabiting deeper soil layers, as the litter layer is more severely affected by management practices than the soil layer, as also evidenced by stronger management effects on microbial community composition of the litter layer (Pollierer et al., 2015). Further, we hypothesized that parthenogenetic Collembola species are more influenced by abiotic factors, whereas sexual ones by biotic drivers such as resource quality. Consequently, we expected populations of parthenogenetic Collembola species to be more abundant in regions with harsh climatic conditions such as the Schorfheide. Further, we expected temporal fluctuations to favor parthenogenetic rather than sexual species as the latter reproduce more quickly.

## 2 Materials and Methods

### 2.1 Study sites

The study sites were part of the “Biodiversity Exploratories,” a large project serving as open platform for biodiversity and ecosystem research (www.biodiversity-exploratories.de; Fischer et al., 2010). The Biodiversity Exploratories cover three regions across Germany, that is, the Swabian Alb, Hainich‐Dün (Hainich), and Schorfheide‐Chorin (Schorfheide). The Schorfheide is a young glacial landscape with an altitude of 3–140 m a.s.l., a mean annual temperature of 8.0–8.5°C and a mean annual precipitation of 500–600 mm. Soils in the Schorfheide are mainly cambisols. The Schorfheide is among the driest landscapes in Germany and has a subcontinental climate with hot and dry summers and cold winters (http://www.schorfheide-chorin.de/seite/109463/landscape.html; Natkhin et al., 2012). The Hainich is based on calcareous bedrock and varies in altitude from 285 to 550 m a.s.l. Soils in the Hainich are mainly luvisols with few stagnosols. The mean annual temperature is 6.5–8.0°C, and the mean annual precipitation is 600–800 mm. The Swabian Alb also has calcareous bedrock, but with karst phenomena. Its soils are cambisols and leptosols. The altitude of the Swabian Alb varies between 460 and 860 m a.s.l., the mean annual temperature is 6–7°C, and the mean annual precipitation is 700–1,000 mm. Acidity of the soil ranged from pH 3.3 ± 0.19 in the Schorfheide to 4.51 ± 0.72 in the Swabian Alb to 4.59 ± 0.67 in the Hainich.

In each region, four forest types with four replicates each were sampled (for details, see Klarner et al., 2014; Pollierer et al., 2015). The forest types included coniferous forests, young even‐aged beech stands (*Fagus sylvatica*; age ~30 years, young managed beech), mature even‐aged beech stands (age ~70 years, old managed beech), and beech stands that were left unmanaged for at least 60 years (age ~120 years, unmanaged beech), representing a gradient of decreasing management intensity. Coniferous forests consisted of spruce (*Picea abies*; age ~60 years) in the Swabian Alb and Hainich, and pine (*Pinus sylvestris*; age ~50 years) in the Schorfheide and were classified as the most intensively managed forests as they had been planted to replace naturally occurring beech forests (Schall & Ammer, 2013). The forest management index (ForMI; Kahl & Bauhus, 2014) was taken from the biodiversity exploratories database (BExIS). The index considers removals and type of dead wood, and the proportion of non‐native conifer species, among other variables. The study sites were located within 100 × 100 m grid plots established as core sampling sites of the Biodiversity Exploratories. The plots were at least 200 m apart from each other and had a minimum distance of 100 m to the next forest edge.

### 2.2 Sampling, extraction, and determination of soil animals

Samples were taken in spring 2008 (May) and 2011 (April and May). In 2008 April featured high precipitation and low temperatures (mean precipitation 30–75 mm, mean temperature 10.0°C), whereas May was unusually warm (mean temperature 14.7°C) and dry, especially in the Schorfheide with only ~10 mm of rain. Spring 2011 was the warmest and driest on record, with great agricultural losses. Soil water content differed between years, depending on region, with similar water contents in the Hainich and Swabian Alb, but significantly lower water content in the Schorfheide at T2 (Pollierer et al., 2015). Soil water content did not differ significantly between forest types, even across the two dates (*F*_3,41_ = 1.44, *p* = .245; repeated‐measures GLM between subject effect for Forest). Samples were taken from 5 × 5 m subplots located within the 100 × 100 m gridplots, as described in Erdmann et al. (2012) and Klarner et al. (2014). Briefly, the four forest types were replicated four times in each region, resulting in 48 sampled forests. In each forest, two soil cores (5 cm Ø) were taken, divided into organic layer (variable thickness) and top 5 cm of soil and mesofauna was extracted by heat (Macfadyen, 1961). Individuals from the two layers were pooled for statistical analyses. Animals were stored in 70% ethanol until identification. Collembola were treated with hydrogen peroxide (one pellet dissolved in ~1 ml distilled water; duration from 30 min to overnight depending on intensity of pigment) and lactic acid (90%; variable duration until individual was transparent) and subsequently mounted on slides for identification under a phase‐contrast microscope using the keys by Gisin (1960), Fjellberg (1998, 2007) and Hopkin (2007).

### 2.3 Statistical analyses

We divided Collembola species into four different life forms (Appendix S1: Table A1), depending on the habitat layer they colonize predominantly. Atmobiotic Collembola inhabit macrophytes such as grasses, bushes, trunks, and branches of trees, but can also be found on the litter surface. Epedaphic Collembola inhabit the upper layer of litter. Hemiedaphic Collembola inhabit litter that is in an advanced stage of decomposition or rotten wood. Euedaphic Collembola mostly inhabit upper mineral layers of soil, but they may also occur higher up in the humus layer. Reproductive mode of the investigated species was inferred from literature (Appendix S1: Table A1). We inferred species to reproduce via parthenogenesis if this reproductive mode has been observed, including species with both parthenogenetic and sexual populations. For some species, there were no literature records on their reproductive mode; in these cases, we inferred it from genus or family level. These species were termed “unknown/parthenogenetic” and “unknown/sexual,” respectively. They were included into the analyses as sexual and parthenogenetic species for sake of completeness. However, omitting these species from the analyses did not significantly change the results of the study (Appendix S1: Table A2).

Environmental parameters of the studied plots, such as pH, C‐to‐N ratios, microbial biomass (C_mic_), and fungal‐to‐bacterial ratios, were taken alongside the mesofauna extraction and have been published in Klarner et al. (2014) and Pollierer et al. (2015). Briefly, microbial biomass was determined using substrate‐induced respiration (SIR; Anderson & Domsch, 1978; Beck et al., 1997) in an automated O_2_ microcompensation apparatus (Scheu, 1992). Phospholipid fatty acids (PLFAs) were extracted from leaf litter as described in Frostegård, Tunlid, and Bååth (1993), and abundances were calculated as nmol per gram dry weight. For the fungal‐to‐bacterial ratios, the PLFA 18:2ω6,9 was used as fungal biomarker, and the PLFAs i15:0, a15:0, i16:0, i17:0, cy17:0, and cy19:0 were chosen to represent bacterial biomass (Frostegård & Bååth, 1996).

Biomass of Collembola was calculated based on mean body length of each species using regressions by Tanaka (1970) and Petersen (1975). Differences in abundances, biomasses, species numbers, life forms, and reproductive mode were analyzed using repeated‐measures general linear models (GLMs), with region (Explo) and forest type (Forest) as between subject effects and time (Date) as within‐subject effect. Differences in community composition were analyzed using discriminant function analysis (DFA) and principal components analysis (PCA).

To investigate fluctuations of Collembola communities between years, the abundance of sexual and parthenogenetic species of the dataset of 2008 was subtracted from those of 2011 for each of the 48 plots. We ignored algebraic signs to only examine the difference between the 2 years, that is, all values were positive. These differences were then expressed as percentage of the mean of the respective group, for example, sexual versus parthenogenetic species per forest type/region. These percentages were log‐transformed if necessary and analyzed by two‐factorial analysis of variance (ANOVA) with the fixed factors region (Schorfheide‐Chorin, Hainich‐Dün, Swabian Alb), and forest type (B30, B70, Bunm, Coniferous).

Repeated‐measures GLMs and ANOVAs were performed using SAS 9.3 (SAS Institute, Cary, NC, USA). Regressions of abundances against environmental parameters and discriminant function analyses (DFA) were performed using STATISTICA 10 (Stat Soft. Inc., 2011, Tulsa, USA). Principal components analyses (PCAs) were performed using Canoco 5 (Ter Braak & Šmilauer, 2012). To assess the main drivers of Collembola total abundances and of abundances of sexual versus parthenogenetic species, we fitted structural equation models using IBM SPSS Amos 20.0 (IBM Corporation Software Group, Somers, NY, USA).

## 3 Results

### 3.1 Abundance and biomass

**Figure 1:**
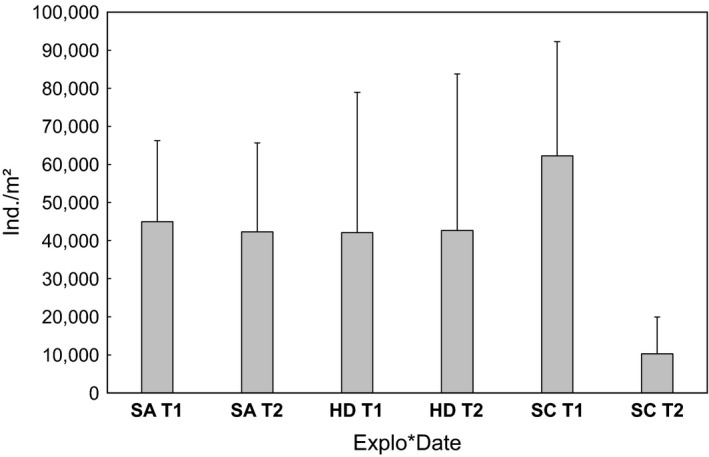
Collembola abundances (individuals per m^2^; means + SD) in the regions Swabian Alb (SA), Hainich‐Dün (HD), and Schorfheide‐Chorin (SC) depending on date (T1—spring 2008, T2—spring 2011)

Collembola abundances differed between years but this varied between regions (*F*_2,36_ = 15.61, *p* < .0001; repeated‐measures GLM within subject effect for Date × Explo; Figure 1), mainly due to pronounced differences in the Schorfheide between T1 and T2 (Table 1). Abundances in the Hainich and the Swabian Alb were similar and rather constant in time (Table 1). Biomasses also differed between years but this again varied between regions (*F*_2,36_ = 5.63, *p* = .0074; repeated‐measures GLM within subject effect for Date × Explo) with pronounced differences between T1 and T2 in the Schorfheide (Table 1), while biomasses in the Swabian Alb and Hainich were similar at T1 and T2 (Table 1). Abundances also differed significantly between the four forest types (*F*_3,36_ = 3.07, *p* = .039; repeated‐measures GLM between subject effect for Forest), with the highest number of individuals in coniferous forests (57,986 ± 44,140 ind./m^2^), and decreasing abundances in the order unmanaged beech forests (39,505 ± 22,859 ind./m^2^), young managed beech forests (33,427 ± 30,141 ind./m^2^), and old managed beech forests (32,101 ± 21,179 ind./m^2^).

**Table 1:** Mean ± standard deviation of Collembola abundance and biomass in the three regions Hainich, Swabian Alb, and Schorfheide in spring 2008 (T1) and 2011 (T2)

	Abundance (ind./m^2^)				Biomass (mg/m^2^)			
	T1		T2		T1		T2	
	Mean	*SD*	Mean	*SD*	Mean	*SD*	Mean	*SD*
Hainich	42,064	36,882	42,661	41,108	104.8	76.3	136.9	244.1
Swabian Alb	44,956	21,300	42,342	23,325	105.3	60.9	106.4	49.5
Schorfheide	62,229	30,018	10,275	9,678	138.8	74.9	22.9	18.8

### 3.2 Diversity

Overall, 99 species of Collembola were recorded. Total species numbers decreased from the Hainich (59 species) to the Schorfheide (49 species) to the Swabian Alb (44 species). The different forest types had similar species numbers; they were highest in coniferous forests (55 species), followed by unmanaged and old managed beech forests (50 species each), and young managed beech forests (48 species). Average species number per sample differed between T1 and T2 (*F*_1,36_ = 102.7, *p* < .0001; repeated‐measures GLM within subject effect for Date), with significantly higher species numbers per sample at T1 (12.2 ± 2.8) than at T2 (6.3 ± 2.7). Species numbers also differed between regions (*F*_2,36_ = 5.22, *p* = .01; repeated‐measures GLM between subject effect for Explo), with lower numbers per sample in the Schorfheide (8.2 ± 4.7) than in the Swabian Alb (9.8 ± 3.7) and Hainich (9.8 ± 3.8). Species numbers per sample also decreased from coniferous forests (10.0 ± 4.2) to unmanaged beech forests (9.3 ± 3.7) and old managed beech forests (9.3 ± 4.2) to young managed beech forests (8.4 ± 4.0). However, this difference was not significant (*F*_3,36_ = 1.90, *p* = .15; repeated‐measures GLM between subject effect for Forest).

### 3.3 Community structure

**Figure 2:**
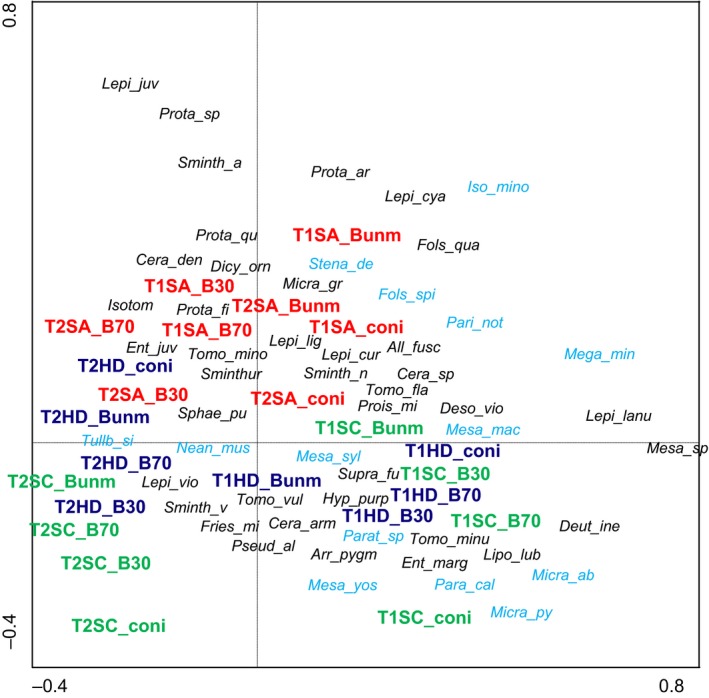
Principal components analysis (PCA) of Collembola species with date (T1—spring 2008, T2—spring 2011), region (SA—Swabian Alb, HD—Hainich‐Dün, SC—Schorfheide‐Chorin) and forest type (B30—young managed beech, B70—old managed beech, Bunm—unmanaged beech, coni—coniferous) as supplementary variables. Parthenogenetic species are marked in blue. For full names of species and abbreviations, see Appendix S1: Table A1

**Figure 3:**
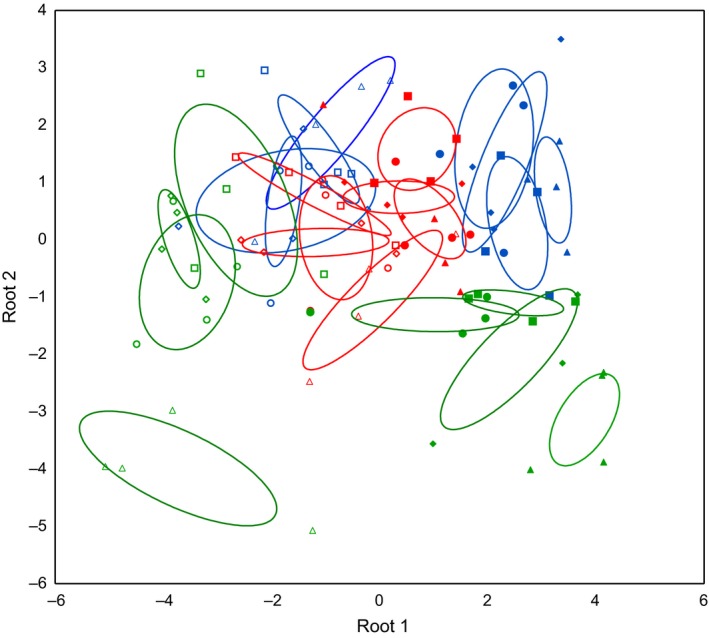
Discriminant function analysis of Collembola community composition (log‐transformed species abundances) in different forest types (diamond—young managed beech, square—old managed beech, circle—unmanaged beech, triangle—coniferous) within the regions Swabian Alb (red), Hainich‐Dün (blue), and Schorfheide‐Chorin (green) in 2008 (filled symbols) and 2011 (open symbols). Ellipses represent confidence intervals at *p* = .05

Principal components analysis (PCA) with date, region, and forest type as supplementary variables separated communities of T1 and T2 along the first axis (eigenvalue 0.14), especially in the Hainich and Schorfheide, and communities of the three regions along the second axis (eigenvalue 0.10), whereas forest types only differed within regions (Figure 2). Supplementary variables accounted for 45.9% of the total variation in species data. These differences were also reflected in discriminant function analysis (*F*_138,398_ = 4.60, *p* < .0001; Figure 3, Table 2). In particular, coniferous forests of the Schorfheide differed markedly between T1 and T2.

**Table 2:** Squared Mahalanobis distances between group centroids and reliability of discrimination for Collembola species composition of coniferous forests (coni), young (B30) and old (B70) managed beech forests, and unmanaged beech forests (Bunm) in the exploratories Swabian Alb (SA), Hainich‐Dün (HD), and Schorfheide‐Chorin (SC), depending on date (T1—spring 2008, T2—spring 2011) . Significant differences are marked in bold

	T2**HD**B30	T2**HD**B70	T2**HD**Bunm	T2**SA**coni	T2**SA**B30	T2**SA**B70	T2**SA**Bunm	T2**SC**coni	T2**SC**B30	T2**SC**B70	T2**SC**Bunm
T2**HD**coni	**8.6** *	5.4	4.8	**12.0** *	5.8	6.1	7.2	**43.9** ***	**15.0** **	**14.4** **	**17.5** **
T2**HD**B30	–	2.5	3.6	**17.1** **	5.9	**16.3** **	**11.5** *	**28.6** ***	**10.6** *	**9.7** *	**15.1** **
T2**HD**B70		–	2.4	**14.5** **	6.1	**12.1** *	**8.3** *	**38.8** ***	**14.5** **	**8.8** *	**17.2** **
T2**HD**Bunm			–	**8.2** *	1.8	6.0	3.2	**25.8** ***	**8.5** *	5.2	**7.6** *
T2**SA**coni				–	5.0	6.9	2.3	**24.6** ***	**20.2** ***	**16.8** **	**13.3** *
T2**SA**B30					–	5.0	1.7	**23.5** ***	**10.5** *	**7.4** *	**8.0** *
T2**SA**B70						–	3.2	**35.9** ***	**20.0** ***	**16.0** **	**13.2** *
T2**SA**Bunm							–	**27.7** ***	**14.7** **	**8.6** *	**9.0** *
T2**SC**coni								–	**19.5** ***	**28.9** ***	**13.7** *
T2**SC**B30									–	6.1	3.8
T2**SC**B70										–	5.7
T2**SC**Bunm											–
T1**HD**coni											
T1**HD**B30											
T1**HD**B70											
T1**HD**Bunm											
T1**SA**coni											
T1**SA**B30											
T1**SA**B70											
T1**SA**Bunm											
T1**SC**coni											
T1**SC**B30											
T1**SC**B70											
T1**SC**Bunm											

	T1**HD**coni	T1**HD**B30	T1**HD**B70	T1**HD**Bunm	T1**SA**coni	T1**SA**B30	T1**SA**B70	T1**SA**Bunm	T1**SC**coni	T1**SC**B30	T1**SC**B70	T1**SC**Bunm
T2**HD**coni	**18.9** ***	**18.0** ***	**21.0** ***	**11.6** *	**11.5** *	4.5	5.1	**12.8** *	**51.7** ***	**27.3** ***	**27.1** ***	**17.0** **
T2**HD**B30	**28.5** ***	**21.9** ***	**21.4** ***	**18.6** ***	**24.9** ***	**18.2** ***	**15.6** **	**30.8** ***	**48.2** ***	**33.6** ***	**26.3** ***	**23.1** ***
T2**HD**B70	**22.8** ***	**13.9** **	**16.6** **	**11.5** *	**18.4** ***	**13.6** *	**8.6** *	**22.6** ***	48.4***	**31.8** ***	**24.6** ***	**19.9** ***
T2**HD**Bunm	**26.5** ***	**19.6** ***	**21.0** ***	**16.5** **	**13.8** *	**10.2** *	**8.4** *	**17.3** **	**46.6** ***	**27.9** ***	**24.6** ***	**15.9** **
T2**SA**coni	**18.7** ***	**17.7** **	**16.1** **	**15.3** **	5.5	5.7	**8.3** *	5.6	**25.4** ***	**8.8** *	**14.3** **	3.2
T2**SA**B30	**20.9** ***	**19.0** ***	**17.5** **	**15.4** **	**9.7** *	**7.4** *	7.1	**13.9** **	**35.1** ***	**20.0** ***	**17.1** **	**12.6** *
T2**SA**B70	**26.2** ***	**26.3** ***	**28.1** ***	**19.8** ***	**10.3** *	4.4	6.3	**7.6** *	**48.3** ***	**26.1** ***	**29.7** ***	**14.9** **
T2**SA**Bunm	**20.2** ***	**16.6** **	**16.9** **	**14.2** **	5.4	5.7	4.8	**7.8** *	**34.6** ***	**17.6** **	**17.0** **	**9.5** *
T2**SC**coni	**72.9** ***	**67.8** ***	**60.1** ***	**65.5** ***	**46.7** ***	**44.2** ***	**52.8** ***	**50.9** ***	**59.1** ***	**43.2** ***	**51.0** ***	**33.2** ***
T2**SC**B30	**50.4** ***	**41.5** ***	**42.0** ***	**38.8** ***	**25.6** ***	**25.7** ***	**27.2** ***	**35.6** ***	**71.2** ***	**44.8** ***	**42.2** ***	**31.7** ***
T2**SC**B70	**40.1** ***	**27.2** ***	**29.0** ***	**28.0** ***	**18.2** ***	**23.7** ***	**17.4** **	**29.9** ***	**59.7** ***	**41.7** ***	**30.6** ***	**29.9** ***
T2**SC**Bunm	**50.8** ***	**42.5** ***	**42.2** ***	40.2****	**20.7** ***	**22.5** ***	**24.3** ***	**27.6** ***	**63.3** ***	**39.5** ***	**39.4** ***	**26.4** ***
T1**HD**coni	–	5.9	4.1	3.2	**13.4** *	**12.6** *	**9.1** *	**16.1** **	**18.4** ***	**11.4** *	**7.2** *	**15.3** **
T1**HD**B30		–	1.9	1.6	**13.0** *	**16.8** **	**8.1** *	**18.5** ***	**26.1** ***	**17.8** **	**9.1** *	**16.0** **
T1**HD**B70			–	3.3	**14.0** **	**17.8** **	**10.6** *	**20.6** ***	**15.3** **	**12.2** *	3.3	**14.2** **
T1**HD**Bunm				–	**11.5** *	**10.2** *	4.7	**14.0** **	**27.2** ***	**15.9** **	**10.7** *	**13.3** *
T1**SA**coni					–	5.9	5.0	4.3	**32.0** ***	**13.0** *	**13.5** *	**10.0** *
T1**SA**B30						–	3.6	3.1	**35.8** ***	**13.9** **	**20.1** ***	**7.7** *
T1**SA**B70							–	6.2	**34.8** ***	**18.4** ***	**15.2** **	**12.3** *
T1**SA**Bunm								–	**35.3** ***	**12.8** *	**22.0** ***	6.9
T1**SC**coni									–	**9.1** *	**7.6** *	**20.3** ***
T1**SC**B30										–	**8.2** *	3.9
T1**SC**B70											–	**14.4** **
T1**SC**Bunm												–

***p < .0001, **p < .001, *p < .05.

Collembola life forms differed between regions and forest types and this often varied between years. The abundance of atmobiotic Collembola differed significantly between forest types (*F*_3,36_ = 3.36, *p* = .0293; repeated‐measures GLM between subject effect for Forest), with highest numbers in coniferous forests (520 ± 821 ind./m^2^), intermediate numbers in natural beech (191 ± 420 ind./m^2^) and young beech forests (159 ± 410 ind./m^2^), and low numbers in old beech forests (53 ± 260 ind./m^2^). Abundance of epedaphic Collembola differed between regions (*F*_2,36_ = 3.63, *p* = .0366; repeated‐measures GLM between subject effect for Explo), with highest numbers in the Hainich (8,240 ± 10,686 ind./m^2^), intermediate numbers in the Swabian Alb (5,568 ± 5,242 ind./m^2^) and lowest numbers in the Schorfheide (3,087 ± 3,359 ind./m^2^). Hemiedaphic Collembola differed significantly between regions but this varied between years (*F*_2,36_ = 5.74, *p* = .0069; repeated‐measures GLM within subject effect for Date × Explo). Abundance was similar in the Swabian Alb at T1 and T2 (15,478 ± 7,967 and 16,161 ± 16,621 ind./m^2^, respectively), whereas in the Hainich, abundance was higher at T2 (24,941 ± 25,379 ind./m^2^) than at T1 (17,501 ± 13,930 ind./m^2^), and in the Schorfheide abundance was considerably lower at T2 (6,013 ± 5,919 ind./m^2^) as compared to T1 (23,526 ± 20,777 ind./m^2^). Abundance of euedaphic Collembola differed between regions but this varied between years (*F*_2,36_ = 9.64, *p* = .0004; repeated‐measures GLM within subject effect for Date × Explo). While abundances were intermediate in the Swabian Alb, and similar at T1 (23,161 ± 13,605 ind./m^2^) and T2 (20,933 ± 14,978 ind./m^2^), in the Hainich and Schorfheide they were higher at T1 than at T2. This was most pronounced in the Schorfheide, with more than 10 times higher numbers at T1 (33,341 ± 17,765 ind./m^2^) than at T2 (2,863 ± 3,584 ind./m^2^). In the Hainich, there were 2.5 times more euedaphic Collembola at T1 (18,533 ± 22,820 ind./m^2^) than at T2 (6,903 ± 9,842 ind./m^2^). Euedaphic Collembola also differed significantly between forest types (*F*_3,36_ = 6.09, *p* = .0018; repeated‐measures GLM between subject effect for Forest), with significantly higher abundance in coniferous forests (27,201 ± 22,128 ind./m^2^) than in beech forests (17,424 ± 16,995, 13,702 ± 17,349, and 12,164 ± 9,721 ind./m^2^ in natural, young, and old beech forests, respectively).

### 3.4 Influence of environmental factors

At T1, abundance (*F*_1,46_ = 15.89, *p* = .0002, *R* ^2^ = .257; regression analysis; Appendix S2: Fig. A1) and biomass (*F*_1,46_ = 14.97, *p* = .0003, *R* ^2^ = .246; regression analysis) were significantly correlated with soil pH, being higher at lower pH. However, when considering sexual and parthenogentic species separately, the negative correlation was only true for abundances of parthenogenetic species (*F*_1,43_ = 18.32, *p* = .0001, *R* ^2^ = .299; regression analysis). Abundance and biomass were also significantly positively correlated with mass of leaf litter (g/cm^2^; abundance: *F*_1,45_ = 12.47, *p* = .001, *R* ^2^ = .217; regression analysis; Appendix S2: Fig. A2; biomass: *F*_1,45_ = 5.22, *p* = .027, *R* ^2^ = .104; regression analysis) and negatively with soil microbial biomass (abundance: *F*_1,46_ = 13.34, *p* = .0007, *R* ^2^ = .225; regression analysis; biomass: *F*_1,46_ = 9.20, *p* = .0039, *R* ^2^ = .167; regression analysis), but were not correlated with leaf litter microbial biomass. Collembola biomass at T1 was correlated with the fungal‐to‐bacterial ratio of leaf litter (*F*_1,42_ = 4.647, *p* = .037, *R* ^2^ = .100; regression analysis), with higher biomass at lower fungal‐to‐bacterial ratios. Abundance and biomass of Collembola were not correlated with C‐to‐N ratio of leaf litter. At T2, abundance, but not biomass, was positively correlated with soil microbial biomass (*F*_1,45_ = 5.43, *p* = .024, *R* ^2^ = .108; Appendix S2: Fig. A3), but neither abundance nor biomass correlated with litter microbial biomass, mass of leaf litter (g/cm^2^), soil pH, fungal‐to‐bacterial ratio of leaf litter nor C‐to‐N ratio of leaf litter.

**Figure 4:**
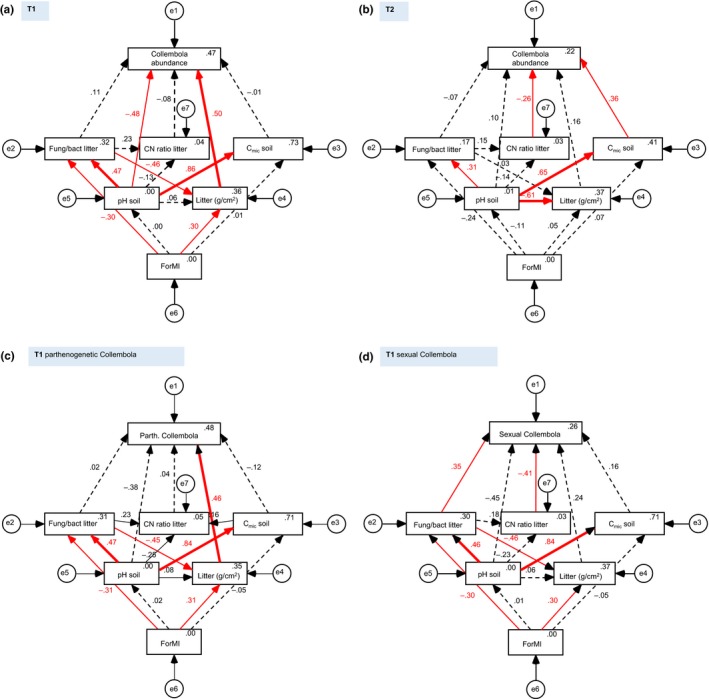
Structural equation models of the drivers of total Collembola abundances in (a) 2008 (T1) and (b) 2011 (T2). If abundances of parthenogenetic (c) and sexual (d) Collembola species are modeled separately for T1, the dependence on abiotic factors is mainly true for parthenogenetic species, whereas sexual species mainly depend on biotic factors and their model resembles that of T2. Rectangles represent observed variables, and circles indicate error terms (e1‐e7). Numbers in rectangles are squared multiple correlations. Red solid arrows indicate significant relationships (thin arrows at *p* < .05, bold arrows at *p* < .001; see also Appendix S1: Table A3). Dotted arrows indicate nonsignificant paths. Numbers on arrows are standardized regression weights; fung/bact litter: fungal‐to‐bacterial ratio of leaf litter, CN ratio litter: C‐to‐N ratio of leaf litter, C_mic_ soil: microbial biomass in soil, litter (g/cm^2^): mass of leaf litter, ForMI: forest management intensity

To test which of the measured environmental factors directly or indirectly influenced Collembola abundance, and how different parameters were interrelated and influenced by forest management intensity (ForMI), we employed structural equation models for T1 and T2 (Figure 4a,b). We achieved the best model fit when including ForMI, pH of soil, mass of leaf litter, fungal‐to‐bacterial ratio and C‐to‐N ratio of leaf litter, and microbial biomass of soil, whereas including microbial biomass and pH of leaf litter, and biomass of earthworms all downgraded the model. At T1 (χ^2^ = 2.1, *df* = 6, probability level: 0.908; for regression weights and levels of significance see Appendix S1: Table A3), Collembola abundance mainly depended on pH of the soil and on leaf litter mass, with a negative influence of pH (i.e., higher abundances at lower pH) and a positive effect of leaf litter mass. In contrast, at T2 (χ^2^ = 2.9, *df* = 6, probability level: 0.825; Appendix S1: Table A3) Collembola abundance was mainly influenced by resource quality, with C‐to‐N ratio of leaf litter and C_mic_ of soil as the main drivers, having a negative and a positive influence, respectively. However, when considering the abundance of sexual and parthenogenetic species separately at T1 (χ^2^ = 2.4, *df* = 6, probability level: 0.875 for only sexual species and χ^2^ = 3.3, df = 5, probability level: 0.659 for only parthenogenetic species; Appendix S1: Table A3), the dependence on abiotic factors was only true for parthenogenetic species (Figure 4c), whereas for sexual species, the model shifted to a main influence of resource quality‐related factors, that is, C‐to‐N ratio of leaf litter and fungal‐to‐bacterial ratio of litter (Figure 4d).

ForMI negatively influenced the fungal‐to‐bacterial ratio of leaf litter and positively the mass of leaf litter; however, this was only significant for T1 (Figure 4a; Appendix S1: Table A3). At T1, the fungal‐to‐bacterial ratio of leaf litter also negatively influenced the mass of leaf litter. At both dates, pH had a positive influence on microbial biomass of soil and on the fungal‐to‐bacterial ratio of litter. At T2, pH additionally had a negative influence on mass of leaf litter (Figure 4b; Appendix S1: Table A3).

### 3.5 Reproductive mode

The abundance of parthenogenetic species differed between years but this varied between regions (*F*_2,36_ = 11.08, *p* = .0002; repeated‐measures GLM within subject effect for Date × Explo). While in the Hainich and Swabian Alb, the abundance of parthenogenetic species was similar at T1 and T2 (25,038 ± 27,103 and 21,505 ± 22,524 ind./m^2^ in the Hainich, and 29,667 ± 18,399 and 26,691 ± 19,217 ind./m^2^ in the Swabian Alb, respectively), the abundance of parthenogenetic species was almost seven times higher in the Schorfheide at T1 (44,603 ± 25,012 ind./m^2^) than at T2 (6,426 ± 7,863 ind./m^2^). This was also mirrored in the interannual fluctuation of the abundance of parthenogenetic Collembola species which differed significantly between regions (*F*_2,36_ = 6.52, *p* = .004), with considerably higher fluctuations in the Schorfheide (150.9 ± 84.8%) than in the Hainich (62.9 ± 75.1%) and Swabian Alb (60.6 ± 64.1%). The abundance of parthenogenetic species also differed significantly between forest types (*F*_3,36_ = 5.65, *p* = .003; repeated‐measures GLM between subject effect for Forest), with highest abundance in coniferous forests (40,392 ± 29,597 ind./m^2^ as compared to 23,266 ± 18,459, 21,496 ± 24,508 and 17,466 ± 10,632 ind./m^2^ in unmanaged, young managed, and old managed beech forests, respectively). Abundance of sexual species did not differ significantly between forest types, but varied significantly between years with the variation depending on region (*F*_2,36_ = 5.03, *p* = .0119; repeated‐measures GLM within subject effect for Date × Explo), with lower numbers in the Schorfheide at T2 (3,849 ± 3,214 ind./m^2^) as compared to T1 (17,626 ± 17,573 ind./m^2^). However, this difference was less pronounced as compared to the abundance of parthenogenetic species. Abundance of sexual species in the Hainich and Swabian Alb was similar in both years (16,978 ± 12,373 and 15,289 ± 8,437 ind./m^2^ at T1, and 21,155 ± 23,126 and 15,652 ± 14,354 ind./m^2^ at T2, respectively). Interannual fluctuations in the abundance of sexual species were also higher in the Schorfheide (129.3 ± 101.3%) than in the Hainich (65.2 ± 54.0%) and Swabian Alb (70.9 ± 61.1%); however, this difference was only marginally significant (*F*_2,36_ = 3.11, *p* = .056).

## 4 Discussion

### 4.1 Temporal and regional differences

Collembola abundance, biomass, and community composition differed markedly between years. Temporal differences were most pronounced in the Schorfheide, and to a lesser extent in the Hainich, whereas communities in the Swabian Alb were rather constant between years. In the Schorfheide, the abundance and biomass of Collembola were six times higher at T1 than at T2, whereas in the Hainich and Swabian Alb, they were similar and constant between years. Principal components analysis and discriminant function analysis separated communities between T1 and T2 along the first axis, especially in the Hainich and Schorfheide, and the three regions along the second axis, whereas differences between forest types were less distinct and only occurred within regions, suggesting that regional factors are more important in structuring Collembola communities than forest management practices. This is in line with the findings of Erdmann et al. (2012) who investigated oribatid mite communities in the same forests. Temporal differences presumably were caused by varying weather conditions between T1 and T2, with T1 featuring moister climate resulting in more favorable conditions such as higher water content in leaf litter and soil and higher microbial biomass (Pollierer et al., 2015). Presumably, regions were affected differently by climatic conditions owing to a north–south gradient, with harsher climatic conditions in the Schorfheide which is characterized by more continental climate, that is, more pronounced differences in temperature and precipitation between seasons. Additionally, the Schorfheide features sandy soils that have lower water holding capacity and therefore are more sensitive to dry weather conditions. Indeed, the water content of soils in the Schorfheide was markedly lower at T2 than at T1, whereas the soil water content in the Hainich and Swabian Alb varied little between the years (Pollierer et al., 2015).

Abundance of life forms differed in time, often depending on region, with the differences being most pronounced in the Schorfheide. Euedaphic Collembola, living in deeper soil layers, were ten times more abundant in the Schorfheide at T1 than at T2, and 2.5 times more abundant in the Hainich at T1. Presumably, the very low abundances in the Schorfheide at T2 were due to the low water holding capacity of Schorfheide soils and thus enhanced detrimental effects of drying on soil living species. Additionally, van Dooremalen, Berg, and Ellers (2013) suggested that euedaphic Collembola are less capable of coping with temperature fluctuations than those living on the surface, presumably due to weaker physiological capabilities such as adjustment of fatty acid composition under changing conditions. Epedaphic Collembola were less abundant in the Schorfheide than in the other two regions, irrespectively of date. Climate more severely affects animals living on the litter surface where they are exposed to higher risk of desiccation and freezing as compared to those living deeper in soil. Consequently, epedaphic Collembola are more affected by the harsh continental climate in the Schorfheide. Abundances of hemiedaphic species were only lower in the Schorfheide at T2, whereas in the Swabian Alb, they did not differ in time and in the Hainich they even were higher at T2. Typically hemiedaphic species colonize both litter and mineral soil suggesting that they are well capable of escaping drought and freezing by retreating into deeper soil layers, thereby being less affected by climatic fluctuations as compared to the other life forms.

Average species numbers differed in time, with almost 50% less species per sample in the dryer year (T2). Presumably higher abundances at T1 increased the likelihood of finding more rare species, that is, increasing species coverage. Again, the Schorfheide had lower species numbers per sample than the other regions. However, total species numbers in the Schorfheide were intermediate between Hainich with the highest and Swabian Alb with the lowest numbers, suggesting that harsh climate is not the primary factor driving diversity of decomposer soil microarthropods. The total species number of Collembola (99) resembled that of Oribatida (114; Erdmann et al., 2012), indicating that the number of niches of both of these decomposer soil microarthropod taxa in the studied forest ecosystems is similar.

### 4.2 Differences between forest types

Collembola communities differed significantly between forest types, with highest abundances and species numbers in coniferous forests. This is similar to abundances of Oribatida (Erdmann et al., 2012), which were at least twice as abundant in coniferous as compared to beech forests. Higher abundance in coniferous forests presumably is due to thicker litter layer as compared to beech forests (Maraun & Scheu, 2000; Salmon et al., 2014); however, humus forms are closely correlated with pH and the presence of macroarthropods such as earthworms (Migge‐Kleian et al., 2006; Schaefer & Schauermann, 1990). Indeed, earthworm abundances were lowest in coniferous forests (M. Ruff, unpubl. data). However, in contrast to Oribatida, which are often negatively influenced by earthworms (Eisenhauer, 2010), Collembola may even benefit from the presence of earthworms (Salmon & Ponge, 2001) that can provide resources and structure in their burrows, thereby enhancing abundances (Loranger et al., 1998). Therefore, other factors, such as soil chemistry/acidity, may have influenced Collembola abundance directly. Ponge (1993) divided Collembola into acidophilic species living in mor, moder or acid mull humus, and neutroacidocline species living in earthworm mull, indicating that the response of species to variations in soil pH differs (Rusek, 1998). However, the effect of pH also may be indirect, for example, via affecting the thickness of organic layers with thick litter layers at low pH favoring decomposer microarthropods (Erdmann et al., 2012; Maraun & Scheu, 2000). High abundances in coniferous forests can also be a consequence of a higher proportion of parthenogenetic species that are adapted to a constant, but resource‐quality poor environment as detailed in the next section. Among beech forests, Collembola abundances were highest in unmanaged forests, indicating that forest management intensity affects Collembola directly through disturbance as suggested by Ponge et al. (2003) or indirectly through altered amounts of resources such as fungi being also reduced by disturbance (de Vries et al., 2009). However, despite a positive influence of ForMI on the mass of leaf litter and a negative influence on the fungal‐to‐bacterial ratio, ForMI did not significantly influence Collembola abundance, suggesting that disturbance via forest management plays a minor role in structuring Collembola communities. It has been suggested that Collembola, assumed to predominantly comprise r‐strategists, recover more quickly from disturbances than, for example, Oribatida, assumed to predominantly comprise K‐strategists (Filser et al., 2002; Maraun & Scheu, 2000). Additionally, differences in species numbers between forest types were rather small. Ponge et al. (2003) also found only small effects of land‐use intensity on total species richness of Collembola.

Principal components analysis suggested Collembola communities to only differ between forest types within regions. Atmobiotic and euedaphic Collembola preferred coniferous forests. Thicker organic layers in coniferous forests (Klarner et al., 2014) may provide more habitat for euedaphic species, whereas atmobiotic species are often phycophages or herbivores feeding on algae and lichens or vascular plants (Chahartaghi et al., 2005). In fact, vascular plants including herbs and bryophytes had the highest species richness in coniferous forests (Boch et al., 2013; Müller et al., 2015). Within beech forests, atmobiotic Collembola had higher numbers in natural and young beech forests, than in old beech forests. Potentially, this was due to higher light intensity in the understory of the former forest types, resulting in a more pronounced cover by herbaceous plants (Boch et al., 2013; Salamon et al., 2008).

### 4.3 Driving factors of Collembola abundance

To identify the driving factors determining Collembola abundance, we considered environmental drivers including soil pH, soil microbial biomass, mass of leaf litter, fungal‐to‐bacterial ratio in leaf litter, leaf litter microbial biomass, and C‐to‐N ratio of leaf litter. While at T1 abundance and biomass were significantly negatively correlated with soil pH, and positively with soil microbial biomass and mass of leaf litter, at T2 abundance was only significantly correlated with soil microbial biomass, with all other correlations being not significant. The negative correlation between soil pH and Collembola abundance contrasts previous studies that even found species assumed to be acidophilic to prefer high pH conditions if having the choice (Hågvar, 1990). However, when considering sexual and parthenogenetic species separately, only parthenogenetic species were significantly negatively correlated with pH. Presumably, high abundance at low pH was due to few acidophilic species such as *Mesaphorura* reproducing via parthenogenesis and benefiting from favorable environmental conditions. Interestingly, while the correlation between abundance and soil microbial biomass was negative at T1, it was positive at T2. This suggests that different climatic conditions in the 2 years caused shifts in the structuring forces of Collembola abundance and community composition.

To assess the relative strength of environmental factors, their influence on Collembola abundance, and their relation to forest management intensity (as reflected by ForMI, see Methods), we applied structural equation modeling (SEM). At T1, SEM suggested Collembola abundance to be mainly driven directly by the mass of leaf litter and soil pH. Leaf litter mass was positively correlated with ForMI and presumably influenced Collembola abundance through provisioning of resources and habitat. Soil pH was positively correlated with the fungal‐to‐bacterial ratio in leaf litter and microbial biomass in soil. The positive correlation between fungal‐to‐bacterial ratio, and soil pH contrasts the assumption that fungi are favored at lower pH (Collins, D'Sylva, & Latter, 1978); however, the relationship between fungal biomass and pH often is weak (Bååth & Anderson, 2003; Lauber et al., 2008). Additionally, Ponge (1993) found no shift in the role of fungal feeding species between acidic and neutro‐alkaline soils. Despite the positive correlation of soil pH with microbial resources, soil pH had a direct negative influence on Collembola abundance, suggesting that at T1 abiotic drivers were more important in driving Collembola abundance than resources. Salamon et al. (2008) also found dominant functional groups/species of Collembola to mainly depend on abiotic factors such as pH and soil water content. In contrast, at T2, SEM suggested Collembola abundance to mainly depend on biotic drivers, such as C‐to‐N ratio of leaf litter and microbial biomass in soil, supporting our conclusion that structuring forces for Collembola communities differed between years. However, when considering the abundance of sexual and parthenogenetic species separately at T1, SEM indicated that the dependence on abiotic factors was only true for parthenogenetic species, whereas for sexual species, the model shifted to a main influence of resource quality‐related factors, that is, C‐to‐N ratio of leaf litter and fungal‐to‐bacterial ratio of litter. This is in line with the assumption that sexual species are superior to parthenogenetic species in exploiting scarce resources and therefore are mainly influenced by resource quality, whereas parthenogenetic species thrive when resources are plenty and abiotic conditions are favorable (Scheu & Drossel, 2007). This may apply in particular to coniferous forests that featured the highest abundances of parthenogenetic Collembola. Parthenogenesis can be an adaptation to a constant, but resource quality poor environment (Petersen, 2002; Scheu & Drossel, 2007), which may have contributed to the high abundance in coniferous forests with thick layers of needle litter being of low food quality due to high lignin content (Taylor, Parkinson, & Parsons, 1989). Bluhm, Scheu, and Maraun (2016) also found the highest numbers of parthenogenetic Oribatida in coniferous forests, and argued that higher amounts of organic material accumulating on the forest floor lead to increased amounts of resources by promoting fungal growth. However, the fungal‐to‐bacterial ratio of leaf litter was lower in coniferous than in beech forests (Pollierer et al., 2015), and the C‐to‐N ratio of leaf litter did not differ significantly between forest types. The “Structured Resource Theory of Sexual Reproduction” (Scheu & Drossel, 2007) states that parthenogenetic species are favored if density‐independent factors, such as desiccation, frost, or flooding, prevail as is the case in the Schorfheide. In the Schorfheide, temporal fluctuations of parthenogenetic Collembola were considerably higher than in the other regions, with seven times higher numbers in the Schorfheide at T1 as compared to T2. Parthenogenetic Oribatida also had highest temporal fluctuations in the Schorfheide, although differences were not as pronounced as for Collembola (Bluhm et al., 2016). Parthenogens can respond more quickly to favorable conditions than sexual populations (Chahartaghi et al., 2009; Maynard‐Smith, 1978) and therefore can better cope with climatic fluctuations. Moist weather conditions at T1 presumably allowed Collembola populations to increase (Salamon et al., 2008), with parthenogenetic Collembola outnumbering sexual ones due to omitting the costs for producing males and other sex associated costs thereby reaching higher abundances within the same time period. Numbers of sexuals also differed between regions depending on time, with a similar pattern as in parthenogens. However, this pattern was not as pronounced and, although temporal fluctuations were most pronounced in the Schorfheide, differences were not significant.

## 5 Conclusions

Collembola abundance, biomass, and community composition differed between years, presumably due to varying weather conditions, resulting in a shift in structuring forces of Collembola communities. Differences were most pronounced in the Schorfheide, the driest region with the most continental climate, presumably favoring parthenogenetic species able to respond more quickly to favorable environmental conditions and to enhanced resource supply. Consistent with this conclusion parthenogenetic Collembola species reached remarkably high abundances in the moister year. Further, SEM supports the conclusion that parthenogenetic species predominantly were controlled by abiotic and density‐independent factors such as pH of soil and mass of leaf litter, whereas sexual species mainly depended on resource‐related factors such as the fungal‐to‐bacterial ratio and the C‐to‐N ratio of leaf litter which likely regulate populations in a density‐dependent way. Differences in Collembola community composition were more pronounced between regions than between forest types, suggesting that regional factors are more important in structuring Collembola communities than management practices or forest types, as also evidenced by the low importance of ForMI.

## Conflict of interest

None declared.
