# Supplementary material for: Driving factors and temporal fluctuation of Collembola communities and reproductive mode across forest types and regions
Source: Ecol Evol. 2017 May 10;7(12):4390–403. doi: 10.1002/ece3.3035 (PMC5478087; doi:10.1002/ece3.3035)
Supplement: Supplementary file 1 [file ECE3-7-4390-s001.pdf]

Table A1 Collembola species with abbreviations used in PCA (Fig. 2), and classification of taxonomic group, life form and reproductive mode (unkn. – unknown; parth. – parthenogenetic).

Species	Abbreviation	Taxonomic group	Life form	Reproductive Mode
Allacma fusca (Linnaeus, 1758)	*All_fusc*	Sminthuridae	atmobiotic	sexual
Arrhopalites pygmaeus (Wankel, 1860)	*Arr_pygm*	Arrhopalitidae	hemiedaphic	sexual
Arrhophalites sp./juv. Börner, 1906		Arrhopalitidae	hemiedaphic	sexual
Ballistura cf. hankoi (Stach, 1959)		Isotomidae	epedaphic	unkn./sexual
Brachystomella parvula (Schaeffer, 1896)		Brachystomellidae	hemiedaphic	unkn./sexual
Ceratophysella armata (Nicolet, 1842)	*Cera_arm*	Hypogastruridae	epedaphic	sexual
Ceratophysella bengtssoni (Ågren, 1904)		Hypogastruridae	epedaphic	sexual
Ceratophysella denticulata (Bagnall, 1941)	*Cera_den*	Hypogastruridae	epedaphic	sexual
Ceratophysella gibbosa (Bagnall, 1940)		Hypogastruridae	epedaphic	sexual
Ceratophysella luteospina (Stach, 1920)		Hypogastruridae	epedaphic	sexual
Ceratophysella sp./juv. Börner, 1932	*Cera_sp*	Hypogastruridae	epedaphic	sexual
Ceratophysella succinea (Gisin, 1949)		Hypogastruridae	epedaphic	sexual
Cryptopygus garretti (Bagnall, 1939)		Isotomidae	epedaphic	sexual
Desoria violacea (Tullberg, 1876)	*Deso_vio*	Isotomidae	epedaphic	sexual [1)]
Deuteraphorura inermis (Tullberg, 1871)	*Deut_ine*	Onychiuridae	euedaphic	sexual [1)]
Deuterosminthurus pallipes (Bourlet, 1842)		Bourletiellidae	epedaphic	unkn./sexual
Deuterosminthurus sp. Borner, 1901		Sminthuridae	epedaphic	unkn./sexual
Dicyrtoma fusca (Lubbock, 1873)		Sminthuridae	atmobiotic	sexual
Dicyrtomina minuta (Nicolet, 1842)		Dicyrtomidae	atmobiotic	sexual
Dicyrtomina ornata (Nicolet, 1842)	*Dicy_orn*	Dicyrtomidae	atmobiotic	sexual
Entomobrya cf. multifasciata (Tullberg, 1871)		Entomobryidae	atmobiotic	sexual
Entomobrya corticalis (Nicolet, 1842)		Entomobryidae	atmobiotic	sexual
Entomobrya marginata /lanuginosa (Tullberg, 1871)	*Ent_marg*	Entomobryidae	atmobiotic	sexual
Entomobrya quinquelineata Börner, 1901		Entomobryidae	atmobiotic	sexual
Entomobryidae juv.	*Ent_juv*	Entomobryidae	atmobiotic	sexual
Folsomia brevicauda Agrell, 1939		Isotomidae	hemiedaphic	sexual
Folsomia fimetaria (Linnaeus, 1758)		Isotomidae	hemiedaphic	sexual
Folsomia juv.		Isotomidae	hemiedaphic	sexual
Folsomia ksenemani Stach, 1947		Isotomidae	hemiedaphic	sexual
Folsomia litsteri (Bagnall, 1939)		Isotomidae	hemiedaphic	sexual
Folsomia quadrioculata (Tullberg, 1871)	*Fols_qua*	Isotomidae	hemiedaphic	sexual
Folsomia spinosa (Kseneman, 1936)	*Fols_spi*	Isotomidae	hemiedaphic	parth. [2)]

Friesea claviseta Axelson, 1900		Neanuridae	epedaphic	sexual
Friesea mirabilis (Tullberg, 1871)	*Fries_mi*	Neanuridae	epedaphic	sexual
Friesea truncata Cassagnau, 1958		Neanuridae	epedaphic	sexual
Gisianus flammeolus (Gisin, 1957)		Katiannidae	epedaphic	unkn./sexual
Hymenaphorura juv. Bagnall 1948		Onychiuridae	euedaphic	unkn./sexual
Hypogastrura burkilli (Bagnall, 1940)		Hypogastruridae	epedaphic	sexual
Hypogastrura purpurascens (Lubbock, 1868)	*Hyp_purp*	Hypogastruridae	epedaphic	unkn./sexual
Hypogastrura sahlbergi (Reuter, 1895)		Hypogastruridae	epedaphic	unkn./sexual
Isotoma hiemalis Schött, 1893		Isotomidae	epedaphic	sexual
Isotoma juv. (cf. viridis)		Isotomidae	epedaphic	sexual
Isotomidae	*Isotom*	Isotomidae	hemiedaphic	unkn./sexual
Isotomiella minor (Schäffer, 1896)	*Iso_mino*	Isotomidae	euedaphic	parth. [3)]
Isotomurus palustris (Muller, 1776)		Isotomidae	epedaphic	sexual
Lepidocyrtus curvicolis Bourlet, 1839	*Lepi_cur*	Entomobryidae	epedaphic	sexual
Lepidocyrtus cyaneus Tullberg, 1871	*Lepi_cya*	Entomobryidae	epedaphic	parth. [3)]
Lepidocyrtus juv. Bourlet, 1839	*Lepi_juv*	Entomobryidae	epedaphic	sexual
Lepidocyrtus lanuginosus (Gmelin, 1788)	*Lepi_lanu*	Entomobryidae	epedaphic	sexual
Lepidocyrtus lignorum (Fabricius, 1793)	*Lepi_lig*	Entomobryidae	epedaphic	sexual
Lepidocyrtus violoceus (Geoffroy, 1762)	*Lepi_vio*	Entomobryidae	epedaphic	sexual
Lipothrix (Sphyrotheca) lubbocki (Tullberg, 1872)	*Lipo_lub*	Sminthuridae	epedaphic	sexual
Megalothorax minimus Willem, 1900	*Mega_min*	Neelidae	euedaphic	parth. [4)]
Mesaphorura italica (Rusek, 1971)		Tullbergiidae	euedaphic	parth. [5)]
Mesaphorura jarmilae Rusek, 1982		Tullbergiidae	euedaphic	unkn./parth.
Mesaphorura macrochaeta Rusek, 1976	*Mesa_mac*	Tullbergiidae	euedaphic	parth. [6)]
Mesaphorura sp. Börner, 1901	*Mesa_sp*	Tullbergiidae	euedaphic	unkn./parth.
Mesaphorura sylvatica (Rusek, 1971)	*Mesa_syl*	Tullbergiidae	euedaphic	parth. [7)]
Mesaphorura tenuisensillata Rusek, 1974		Tullbergiidae	euedaphic	parth. [8)]
Mesaphorura yosii (Rusek, 1967)	*Mesa_yos*	Tullbergiidae	euedaphic	unkn./parth.
Metaphorura affinis (Börner, 1903)		Tullbergiidae	euedaphic	parth. [6)]
Micanurida forsslundi Gisin, 1949		Neanuridae	euedaphic	unkn./sexual
Micranurida cf. sensillata (Gisin, 1953)		Neanuridae	euedaphic	unkn./sexual
Micranurida granulata (Agrell, 1943)	*Micra_gr*	Neanuridae	euedaphic	unkn./sexual
Micranurida juv. Börner, 1901		Neanuridae	euedaphic	unkn./sexual
Micranurida pygmaea Börner, 1901	*Micra_py*	Neanuridae	euedaphic	parth. [9)]
Micraphorura absoloni (Börner, 1901)	*Micra_ab*	Onychiuridae	euedaphic	parth. [10), 11)]
Neanura muscorum (Templeton, 1835)	*Nean_mus*	Neanuridae	hemiedaphic	parth. [3)]
Neelides minutus (Folsom, 1901)		Neelidae	euedaphic	parth. [12)]
Neonaphorura duboscqi (Denis, 1932)		Tullbergiidae	euedaphic	unkn./sexual

Neotullbergia ramicuspis (Gisin, 1953)		Onychiuridae	euedaphic	sexual
Oncopodura crassicornis Shoebotham, 1911		Oncopoduridae	euedaphic	unkn./sexual
Orchesella bifasciata (Bourlet, 1839)		Entomobryidae	atmobiotic	sexual
Orchesella cf. flavescens (Bourlet, 1839)		Entomobryidae	atmobiotic	parth. [3)]
Paratullbergia callipygos (Börner, 1902)	*Para_cal*	Tullbergiidae	euedaphic	parth. [6), 11)]
Paratullbergia macdougalli Bagnall, 1936		Tullbergiidae	euedaphic	unkn./parth.
Paratullbergia sp. Womersley, 1930	*Parat_sp*	Tullbergiidae	euedaphic	unkn./parth.
Parisotoma notabilis (Schaeffer, 1896)	*Pari_not*	Isotomidae	hemiedaphic	parth [3)]
Proisotoma minima (Absolon, 1901)		Isotomidae	epedaphic	parth [9)]
Proisotoma minuta (Tullberg, 1871)	*Prois_mi*	Isotomidae	epedaphic	sexual [1)]
Protaphorura armata (Tullberg, 1869)	*Prota_ar*	Onychiuridae	euedaphic	parth [5)]
Protaphorura aurantiaca (Ridley, 1880)		Onychiuridae	euedaphic	sexual
Protaphorura fimata (Gisin, 1952)	*Prota_fi*	Onychiuridae	euedaphic	sexual
Protaphorura quadriocellata (Gisin, 1947)	*Prota_qu*	Onychiuridae	euedaphic	sexual
Protaphorura sp. Absolon, 1901	*Prota_sp*	Onychiuridae	euedaphic	sexual
Pseudachorutes cf. dubius ***K***rausbauer, 1898		Neanuridae	epedaphic	sexual
Pseudachorutes subcrassus Tullberg, 1871		Neanuridae	epedaphic	sexual
Pseudanurophorus binoculatus Kseneman, 1934		Isotomidae	hemiedaphic	parth. [1)]
Pseudosinella alba (Packard, 1873)	*Pseud_al*	Entomobryidae	euedaphic	sexual
Pseudosinella decipiens Denis, 1924		Entomobryidae	euedaphic	unkn./sexual
Pseudosinella immaculata (Lie-Pettersen, 1896)		Entomobryidae	euedaphic	unkn./sexual
Pseudosinella ksenemani /denisi (Gisin, 1954)		Entomobryidae	euedaphic	unkn./sexual
Sminthuridae juv.		Sminthurididae	epedaphic	unkn./sexual
Sminthurides sp. Börner, 1900		Sminthurididae	epedaphic	unkn./sexual
Sminthurinus aureus (Lubbock, 1862)	*Sminth_a*	Katiannidae	epedaphic	sexual/both
Sminthurinus niger (Lubbock, 1868)	*Sminth_n*	Katiannidae	epedaphic	sexual [3)]
Sminthurinus sp. juv. Börner, 1901	*Sminthur*	Katiannidae	epedaphic	unkn./sexual
Sminthurus viridis (Linnaeus, 1758)	*Sminth_v*	Sminthuridae	epedaphic	unkn./sexual
Sphaeridia pumilis (da Gama, 1964)	*Sphae_pu*	Sminthurididae	epedaphic	sexual [3)]
Stenaphorura denisi (Bagnall, 1935)	*Stena_de*	Tullbergiidae	euedaphic	parth [7), 11)]
Stenaphorura quadrispina (Börner, 1901)		Tullbergiidae	euedaphic	sexual
Supraphorura furcifera (Börner, 1901)	*Supra_fu*	Onychiuridae	euedaphic	unkn./sexual
Tomocerus baudoti Denis, 1932		Tomoceridae	epedaphic	sexual
Tomocerus flavescens (Tullberg, 1871)	*Tomo_fla*	Tomoceridae	epedaphic	sexual
Tomocerus minor (Lubbock, 1862)	*Tomo_mino*	Tomoceridae	epedaphic	sexual

Tomocerus minutus (Tullberg, 1876)	*Tomo_minu*	Tomoceridae	epedaphic	sexual
Tomocerus vulgaris (Tullberg, 1871)	*Tomo_vul*	Tomoceridae	epedaphic	sexual
Tullbergia simplex	*Tulb_si*	Tullbergiidae	euedaphic	parth [6)]
Willemia anophthalma Börner, 1901		Hypogastruridae	euedaphic	parth [9)]
Willemia aspinata Stach, 1949		Hypogastruridae	euedaphic	parth [3)]
Xenylla sp. Tullberg, 1869		Hypogastruridae	epedaphic	unkn./sexual
Xenylla grisea Axelson, 1900		Hypogastruridae	epedaphic	sexual [13)]

References for reproductive mode:

[1)] Santorufo, L. et al. 2014. An assessment of the influence of the urban environment on collembolan communities in soils using taxonomy- and trait-based approaches. - Appl. Soil Ecol. 78: 48–56.

[2)] Fjellberg, A. 2007. The Collembola of Fennoscandia and Denmark. Part II: Entomobryomorpha and Symphy- pleona. - Fauna Entomol. Scand. 42: 1–263.

[3)] Chahartaghi, M. et al. 2006. Sex ratio and mode of reproduction in Collembola of an oak-beech forest. - Pedobiologia 50: 331–340.

[4)] Petersen, H. 1971. Parthenogenesis in two common species of Collembola: *Tullbergia krausbaueri* (Börner) and *Isotoma notabilis* Schäffer. - Rev. Ecol. Biol. Sol, 8: 133–138.

[5)] Czarnetzki, A. B. and Tebbe, C. C. 2004. Diversity of bacteria associated with Collembola - A cultivation- independent survey based on PCR-amplified 16S rRNA genes. - FEMS Microbiol. Ecol. 49: 217–227.

[6)] Fjellberg, A. 1998. The Collembola of Fennoscandia and Denmark. Part I: Poduromorpha. - Fauna Entomol. Scand. 35: 1–183 (1998).

[7)] Petersen, H. 1980. Population dynamic and metabolic characterization of Collembola species in a beech forest ecosystem. In: Dindal, D. L. (ed) Soil biology as related to land use practices. Proceedings of the VII International Soil Zoology Colloquium of the I.S.S.S., Syracuse, N.Y., U.S.A. July 29 Aug. 3, 1979. EPA560/1380038, Washington D.C., pp. 806–833.

[8)] Russell, D.J., Hohberg, K. and Pfanz, H. 2011. Occurrence of collembolan fauna in mofette fields (natural carbon- dioxide springs) of the Czech Republic. - Soil Org. 83: 489–505.

[9)] Chernova, N. M. et al. 2010. Ecological significance of parthenogenesis in collembola. - Entomol. Rev. 90: 23–38.

[10)] Pomorski, R.J. 1998. Onychiurinae of Poland (Collembola: Onychiuridae). - Genus Suppl. 9: 1–201.

[11)] Petersen, H. 1978. Sex-ratios and the extent of parthenogenetic reproduction in some collembolan populations. First International Seminar on Apterygota. Accademia delle Scienze di Siena detta de'Fisiocritici, Siena.

[12)] Schneider, C. et al. 2011. Unexpected diversity in Neelipleona revealed by molecular phylogeny approach (Hexapoda, Collembola). - Soil Org. 83: 383–398.

[13)] Sharma, G. D. and Metz, L. J. 1976. Biology of the Collembola *Xenylla grisea* Axelson and *Lepidocyrtus cyaneus* f. *cinereus* Folsom. - Ecol. Entomol. 1: 209-212.

References for classification of life form

Christiansen, K. 1964. Bionomics of Collembola. - Annu. Rev. Entomol. 9: 147-178.

Rusek, J., 2007. A new classification of Collembola and Protura life forms. - In: Tajovský, K. et al. (eds.), Contributions to soil zoology in Central Europe II. pp. 109-115.

Table A2 Repeated measures GLM for differences in the abundance of parthenogenetic and sexual Collembola as influenced by region (Explo), forest type (Forest) and year (Date). Parthenogenetic – species that were observed to have parthenogenetic populations; Sexual – species with sexual reproduction; Complete parth. and Complete sex. - including "unknown/parthenogenetic" and "unknown/sexual" species, respectively, from Table A1. Significant effects are marked in bold.

		Parthenogenetic		Sexual		Complete parth.		Complete sex.	
	DF	F	p	F	p	F	p	F	p
**Between subject effects**									
Explo	2	1.08	0.351	1.82	0.177	0.44	0.645	1.98	0.153
Forest	3	**4.05**	**0.014**	0.56	0.647	**5.65**	**0.003**	0.51	0.681
Explo*Forest	6	1.18	0.341	1.58	0.181	0.87	0.527	1.51	0.204
Error	36								
**Within subject effects**									
Date	1	**9.59**	**0.004**	1.18	0.285	**18.14**	**0.0001**	1.60	0.214
Date*Explo	2	**7.65**	**0.002**	**5.55**	**0.008**	**11.08**	**0.0002**	**5.03**	**0.012**
Date*Forest	3	1.02	0.397	1.30	0.290	2.33	0.091	1.30	0.289
Date*Explo*Forest	6	0.58	0.741	0.56	0.758	0.92	0.492	0.53	0.783
Error	36								

Table A3 Regression weights for paths in structural equation models for Collembola abundance at a) T1 and b) T2 and c) and d) for abundance of only parthenogenetic and sexual Collembola at T1, respectively. Estimate – estimate of regression weight, S.E. – standard error for estimate, C.R. – critical ratio for regression weight, P – level of significance for regression weight, *** - P< 0.001. For the respective structural equation models see Fig. 4 a), b), c) and d).

**a) T1**			Estimate	S.E.	C.R.	P
pH soil	←	ForMI	.005	.181	.027	.979
fung/bact litter	←	ForMI	-.718	.293	-2.447	.014
fung/bact litter	←	pH_soil	.924	.244	3.791	***
CN ratio litter	←	fung/bact litter	.784	.573	1.368	.171
litter (g/cm^2)	←	ForMI	.141	.060	2.338	.019
litter (g/cm^2)	←	pH soil	.025	.054	.464	.642
litter (g/cm^2)	←	fung/bact litter	-.091	.029	-3.139	.002
CN ratio litter	←	pH soil	-.844	1.121	-.753	.452
C_{mic} soil	←	pH soil	513.449	46.805	10.970	***
C_{mic} soil	←	ForMI	6.270	56.334	.111	.911
summe_ind	←	fung/bact litter	2125.608	2813.986	.755	.450
summe_ind	←	litter (g/cm^2)	47931.214	12488.027	3.838	***
summe_ind	←	C_{mic} soil	-.378	13.162	-.029	.977
summe_ind	←	pH soil	-18051.671	8242.627	-2.190	.029
summe_ind	←	CN ratio litter	-446.179	622.902	-.716	.474

**b) T2**			Estimate	S.E.	C.R.	P
pH soil	←	ForMI	-.144	.193	-.747	.455
fung/bact litter	←	ForMI	-.355	.200	-1.771	.077
fung/bact litter	←	pH soil	.351	.150	2.344	.019
CN ratio litter	←	fung/bact litter	.813	.826	.984	.325
litter (g/cm^2)	←	ForMI	.006	.017	.376	.707
litter (g/cm^2)	←	pH_soil	-.065	.013	-5.006	***
CN ratio litter	←	pH soil	-.839	1.145	-.732	.464
C_{mic} soil	←	pH soil	445.878	78.076	5.711	***
C_{mic} soil	←	ForMI	67.753	104.260	.650	.516
litter (g/cm^2)	←	fung/bact litter	.003	.012	.262	.793
summe_ind	←	litter (g/cm^2)	52085.806	53425.213	.975	.330
summe_ind	←	C_{mic} soil	18.077	8.591	2.104	.035
summe_ind	←	pH soil	3581.515	7084.716	.506	.613
summe_ind	←	fung/bact litter	-2111.746	4321.431	-.489	.625
summe_ind	←	CN ratio litter	-1501.520	753.980	-1.991	.046

**c) T1 parth.**			Estimate	S.E.	C.R.	P
pH soil	←	ForMI	.019	.175	.106	.916
fung/bact litter	←	ForMI	-.717	.289	-2.481	.013
fung/bact litter	←	pH soil	.914	.240	3.807	***
C_{mic} soil	←	pH soil	526.086	49.066	10.722	***
C_{mic} soil	←	ForMI	-38.905	59.084	-.658	.510
CN ratio litter	←	fung/bact litter	.783	.571	1.372	.170
litter (g/cm^2)	←	ForMI	.139	.058	2.410	.016
litter (g/cm^2)	←	pH_soil	.029	.052	.565	.572
litter (g/cm^2)	←	fung/bact litter	-.087	.028	-3.053	.002

CN ratio litter	←	pH soil	-1.660	1.828	-.908	.364
CN ratio litter	←	C_{mic} soil	.002	.003	.602	.547
Parth Coll.	←	fung/bact litter	379.930	2211.579	.172	.864
Parth Coll.	←	litter (g/cm^2)	36104.836	9730.269	3.711	***
Parth Coll.	←	C_{mic} soil	-5.622	9.249	-.608	.543
Parth Coll.	←	pH soil	-11153.273	6038.875	-1.847	.065
Parth Coll.	←	CN ratio litter	165.826	475.213	.349	.727

**d) T1 sex.**			Estimate	S.E.	C.R.	P
pH soil	←	ForMI	.012	.177	.066	.947
fung/bact litter	←	ForMI	-.707	.295	-2.399	.016
fung/bact litter	←	pH soil	.885	.243	3.641	***
CN ratio litter	←	fung/bact litter	.613	.566	1.083	.279
litter (g/cm^2)	←	ForMI	.138	.057	2.413	.016
litter (g/cm^2)	←	pH soil	.022	.051	.430	.667
litter (g/cm^2)	←	fung/bact litter	-.089	.028	-3.226	.001
CN ratio litter	←	pH soil	-.593	1.082	-.548	.584
C_{mic} soil	←	pH soil	526.692	48.708	10.813	***
C_{mic} soil	←	ForMI	-39.182	59.046	-.664	.507
Sexual Coll.	←	fung/bact litter	2946.534	1419.855	2.075	.038
Sexual Coll.	←	litter (g/cm^2)	10190.887	6463.087	1.577	.115
Sexual Coll.	←	C_{mic} soil	4.096	6.046	.678	.498
Sexual Coll.	←	pH soil	-7170.213	3911.652	-1.833	.067
Sexual Coll.	←	CN ratio litter	-1004.070	308.113	-3.259	.001
