# Supplementary material for: Driving factors and temporal fluctuation of Collembola communities and reproductive mode across forest types and regions
Source: Ecol Evol. 2017 May 10;7(12):4390–403. doi: 10.1002/ece3.3035 (PMC5478087; doi:10.1002/ece3.3035)
Supplement: Supplementary file 2 [file ECE3-7-4390-s002.pdf]

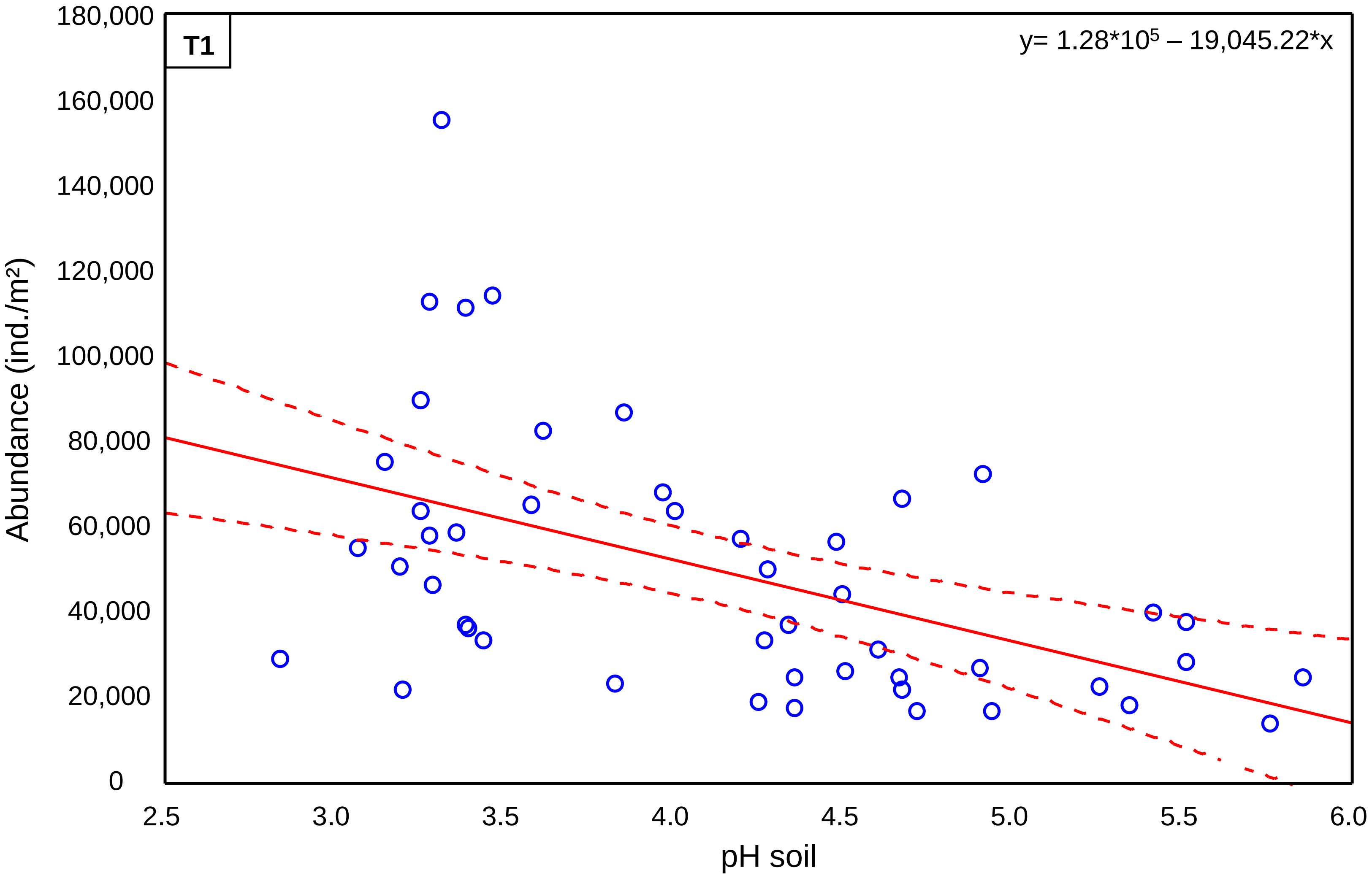
T1
y= 1.28*10⁵ – 19,045.22*x
Abundance (ind./m²)
180,000
160,000
140,000
120,000
100,000
80,000
60,000
40,000
20,000
0
2.5
3.0
3.5
4.0
4.5
5.0
5.5
6.0
pH soil

Fig. A1

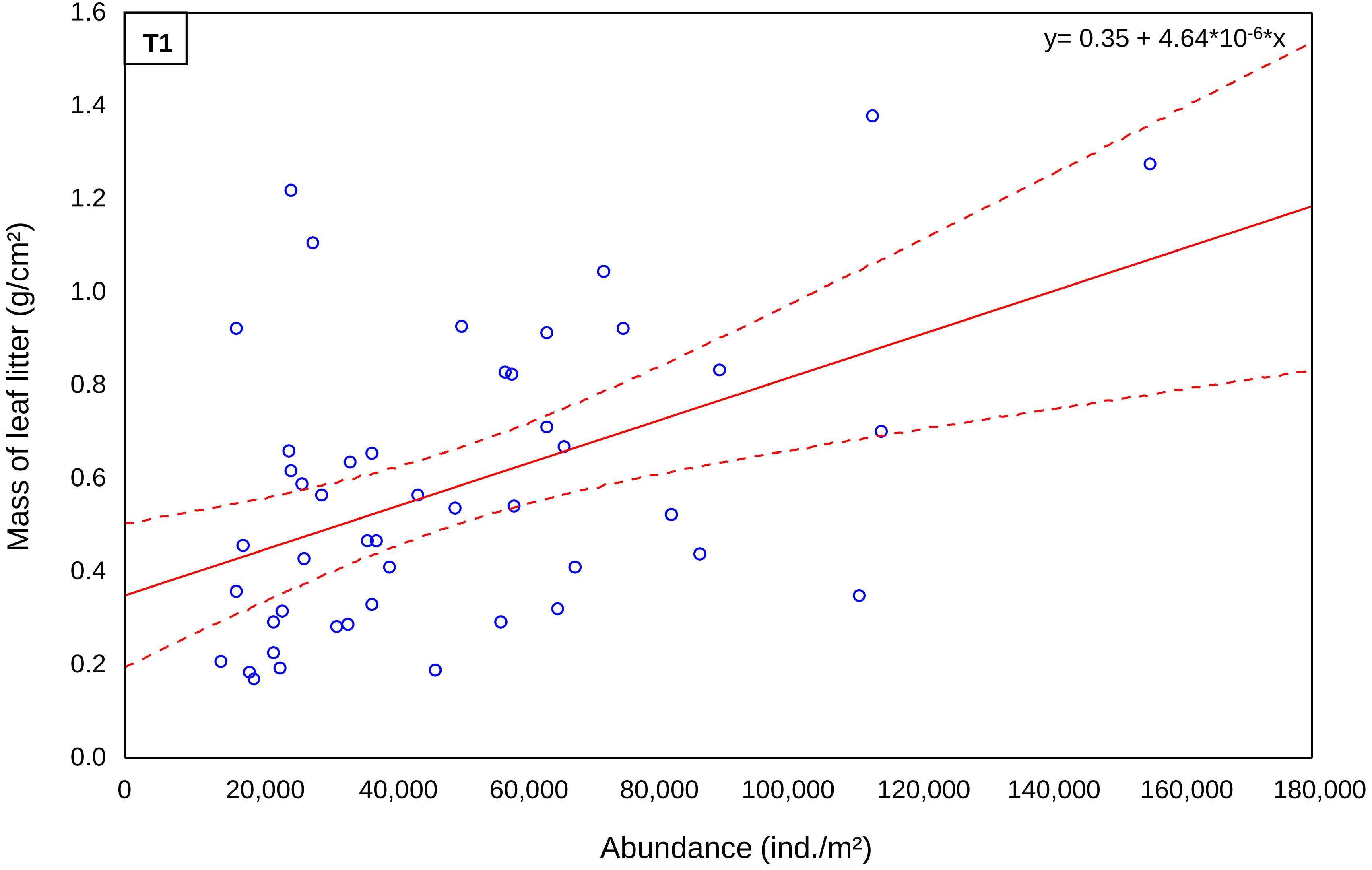
T1
y= 0.35 + 4.64*10-6*x
Mass of leaf litter (g/cm²)
Abundance (ind./m²)
1.6
1.4
1.2
1.0
0.8
0.6
0.4
0.2
0.0
0
20,000
40,000
60,000
80,000
100,000
120,000
140,000
160,000
180,000

Fig. A2

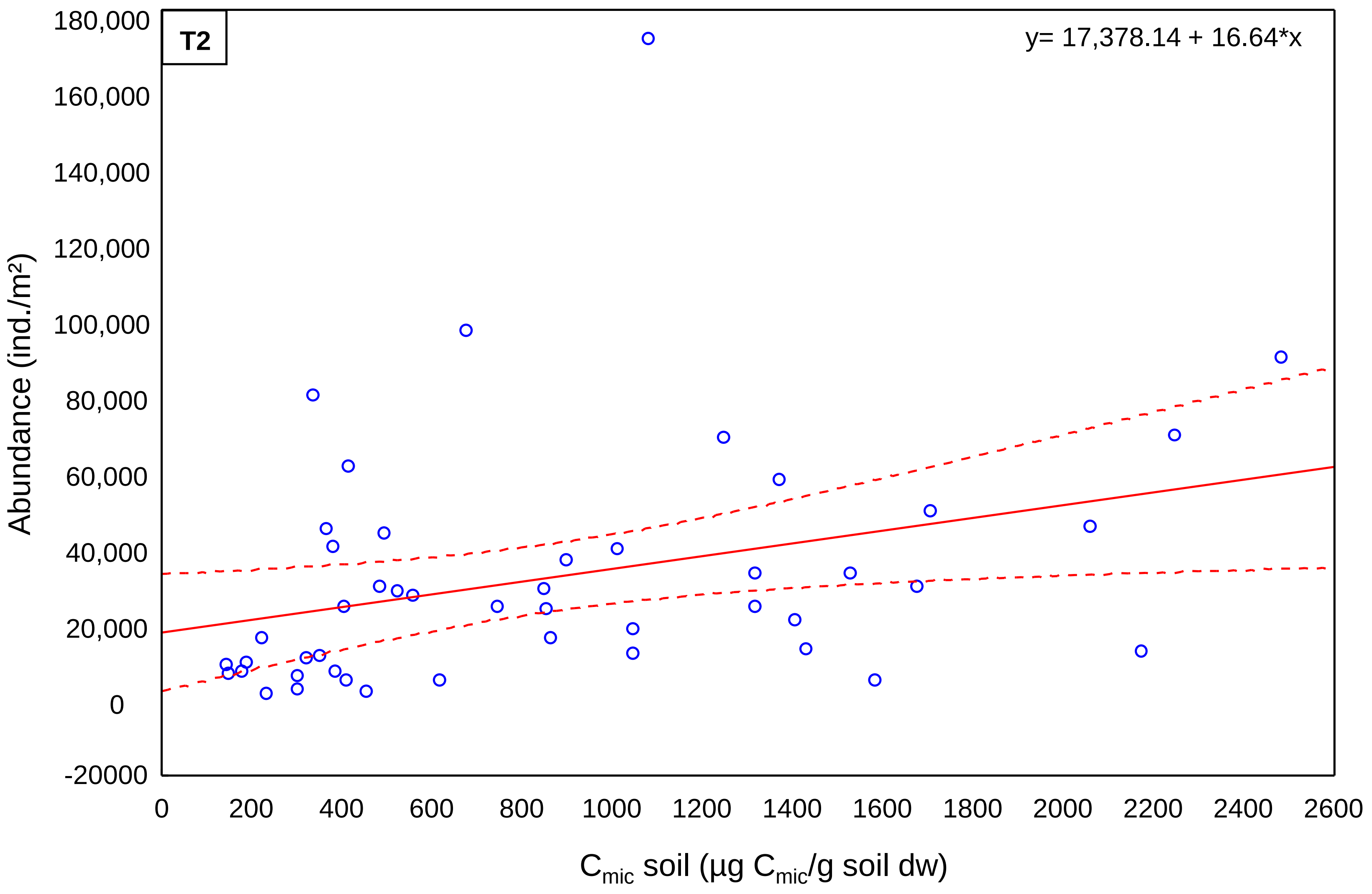
T2
y= 17,378.14 + 16.64*x
Abundance (ind./m²)
180,000
160,000
140,000
120,000
100,000
80,000
60,000
40,000
20,000
0
-20000
0
200
400
600
800
1000
1200
1400
1600
1800
2000
2200
2400
2600
Cmic soil (µg Cmic/g soil dw)

Fig. A3

Legends to figures in Supplementary material Appendix 2

Fig. A1 Correlation between pH of soil and the abundance of Collembola (individuals per m^2) in 2008 (T1). Regression bands represent 95 % confidence intervals.

Fig. A2 Correlation between abundance of Collembola (individuals per m^2) and mass of leaf litter (g/cm^2) in 2008 (T1). Regression bands represent 95 % confidence intervals.

Fig. A3 Correlation between microbial biomass of soil (Cmic soil) and abundance of Collembola (individuals per m^2) in 2011 (T2). dw: dry weight. Regression bands represent 95 % confidence intervals.
